# Supplementary material for: Patterns of Intron Gain and Loss in Fungi
Source: PLoS Biol. 2004 Nov 30;2(12):e422. doi: 10.1371/journal.pbio.0020422 (PMC532390; doi:10.1371/journal.pbio.0020422)
Supplement: Table S1 — Also available at http://genes.mit.edu/NielsenEtAl/. (4.3 MB ZIP). [file pbio.0020422.st001.zip › NielsenEtAl/html/1279.html]

AN0057.1.NCU07755.1.MG02449.1.FG07436.1


```
 CLUSTAL W (1.82) Multiple Sequence Alignments - Introns Inserted


Sequence 1: AN0057.1	397 aa
Sequence 2: FG07436.1	398 aa
Sequence 3: NCU07755.1	409 aa
Sequence 4: MG02449.1	391 aa
Alignment Length: 426 aa
Number Identitical Residues: 201 aa
Alignment Score (without introns) 9505


MG02449.1 	--------------------MASLSKEERLALIKENLEEHLNIEIIEDILAQGRDPKIYW
NCU07755.1	MTTDSTLAATADAKLAISGDNTKLTKEQQLALINVNLAEILNPEIIEKVIDEGRAPRIYW
FG07436.1 	---------------------MALSNEERLGLIKENLAEVLNPEIIEGILAEGRSPKIYW
AN0057.1  	---------------------MDAAAKARFDLITENLAETLNPEIIESILAEGRNPRIYW
          	                        : : :: **. ** * ** **** :: :** *:***

MG02449.1 	G1TATTG~RPHIGYFVPALKIAQFLAAGCELTILLADIHGFLDNLKAPIEIVQYRAQYYQ
NCU07755.1	G1TATTG1RPHCGYLVPAIKIAQLLAAGCHVIVLLADIHAVLDNLKSTVELVGYRAKYYE
FG07436.1 	G1TATTG~RPHCGYLVPAVKIAQYLAAGCEVTVLLADIHGFLDNLKAPIELVEKRAEYYK
AN0057.1  	G~TATTG~RPHTGYFVAAIKIAQLLAAGCEVVVLLADIHAFLDNLKAPLELVENRAKYYA
          	* ***** *** **:*.*:**** *****.: :******..*****:.:*:*  **:** 

MG02449.1 	HVIKSILKAVGVPTEKLRFILGSEFQKGPDYIMDIYKLTSLTSEHDARKAGAEVVKQTDN
NCU07755.1	RTITALLKAVGVPTDKLEFVLGSSYQKSPDYIMDVYKLATLVSEHDAKKAGAEVVKQSDN
FG07436.1 	FVVESMLKSCGVSTEQLRFVLGSSYQKTSGYVMDLYRLSSVVSEHDAKKAGAEVVKQTGN
AN0057.1  	KVIRAILESVGVPTEKLEFVLGSSYQKTPEYTMDVYKLSSLVSEHDAKKAGAEIVKQSAN
          	 .: ::*:: **.*::*.*:***.:** . * **:*:*:::.*****:*****:***: *

MG02449.1 	APLSGLLYPLLQALDEEYLGCDAQFGGTDQRKIFTLAKDVLPKIGYKQ~RAHLMNPMIPG
NCU07755.1	APLSGLLYPILQVLDEEYLKVDAQFGGVDQRKLFAAAKDWLPRLGYKE0RAHIMNPMVPG
FG07436.1 	APLSGLMYPLLQVLDEQYLDCDIQFGGVDQRKLFAAATEWLPKLGYRK0RAHLMNPMVAG
AN0057.1  	APLSGLLYSILQVLDEEHLKVDAQLGGMDQRKLFAAAVEWLPKIGYRK0RAHLVTPMVAG
          	******:*.:**.***::*  * *:** ****:*: * : **::**:: ***::.**:.*

MG02449.1 	L0QGAKMSSSEPA1SSD~SKIDLLDSAEAVAKKLRKAEAVPKVPDGNGIIAFIEYVL2LP
NCU07755.1	L~QGSKMSSSDP-~--D~SKIDLLDAPDVVTKKIRKAEAAPKVTEGNGLISFIEYVL~LP
FG07436.1 	L~NGNKMSSSDE-~--N1SKIDLLDSPEAVTKKIRKAECVPKEVEGNGVLALVEYVL~LP
AN0057.1  	L~SGGKMSSSVQ-~--D1SKIDLLDGPDVIQKKIRKAEAAPKVVEDNGVIAMVEYVL~LP
          	* .* *****      : *******..:.: **:****..**  :.**:::::**** **

MG02449.1 	ASKLKTGRQEFHVERERDGLEPLVYNDIAKLREDYKNDV0LTPQLLKPATTKALLELLTP
NCU07755.1	AAQLL-GKEGFRVDRSRDNLEPLVYTDIAKIKADYESDV0LTPQLVKPAISAAINELLAP
FG07436.1 	ASGLRTGTREFKVER-RD-AEPLVYSDINKVHEDYRNDV0LTPQTLKAAVTEGILALMNP
AN0057.1  	AAGLK-GKKEFVVER-RD-AEPLVYTDIAKLKEDYANDI~LTPQALKPAVTAALTSLMAP
          	*: *  * . * *:* **  *****.** *:: ** .*: **** :*.* : .:  *: *

MG02449.1 	IQAEFEASKEWQEVTEKAYPPEEKKKKEKKVKNKGTRHPGAAG--------------NAP
NCU07755.1	IQAAFQASPEWQEIATKAYPPEIKVKKEKKVKNKGTRYPGAAAKL-----------EDAK
FG07436.1 	IQADYQASKEWQEVTLKAYPPPVK--KEKKVKNLGTRFPGAKGGKGGDAAEVTKKAEDLT
AN0057.1  	IQAAYQASPEWQEITAQAYPPPVVEKKQKKVKSKGTRYPGAQAQAQAQAEAQAEVEGAGK
          	*** ::** ****:: :****    .*:****. ***.*** .   ..:   :.      

MG02449.1 	AETLPERPKETMA-
NCU07755.1	DEATPEPATENA--
FG07436.1 	IKDVQEQSPANPTA
AN0057.1  	EQELPERPKAEN--
          	 :   * .
```
